# Supplementary material for: Genome-wide CRISPR Screens in T Helper Cells Reveal Pervasive Crosstalk between Activation and Differentiation
Source: Cell. 2019 Feb 7;176(4):882–896.e18. doi: 10.1016/j.cell.2018.11.044 (PMC6370901; doi:10.1016/j.cell.2018.11.044)
Supplement: Data S2. Processed Data from All the Steps of the Analysis, Related to Figure 1 [file mmc2.zip › supplemental data/motif analysis/Bhlhe40_homer/homerResults/motif3.similar.html]

motif3

## Information for motif3

C
A
G
T
G
C
T
A
T
C
A
G
C
T
A
G
A
G
T
C
G
A
T
C
T
G
A
C
A
T
G
C
C
T
A
G
G
A
T
C
T
A
G
C
A
G
T
C
  
Reverse Opposite:  

C
T
A
G
A
T
C
G
C
T
A
G
G
A
T
C
T
A
C
G
A
C
T
G
C
T
A
G
T
C
A
G
G
A
T
C
A
G
T
C
C
G
A
T
G
T
C
A
  

|  |  |
| --- | --- |
| p-value: | 1e-28 |
| log p-value: | -6.600e+01 |
| Information Content per bp: | 1.652 |
| Number of Target Sequences with motif | 283.0 |
| Percentage of Target Sequences with motif | 23.58% |
| Number of Background Sequences with motif | 5065.0 |
| Percentage of Background Sequences with motif | 11.91% |
| Average Position of motif in Targets | 98.3 +/- 53.9bp |
| Average Position of motif in Background | 87.3 +/- 63.4bp |
| Strand Bias (log2 ratio + to - strand density) | 0.3 |
| Multiplicity (# of sites on avg that occur together) | 1.44 |
| Motif File: | file (matrix) reverse opposite |

### Similar de novo motifs found

|  |  |  |  |  |  |  |  |
| --- | --- | --- | --- | --- | --- | --- | --- |
| Rank | Match Score | Redundant Motif | P-value | log P-value | % of Targets | % of Background | Motif file |
| 1 | 0.948 | C A T G A G T C A G T C A G T C A G T C A C T G A G T C A G T C A G T C G A T C | 1e-26 | -61.838904 | 22.42% | 11.36% | motif file (matrix) |
| 2 | 0.918 | G A T C A G C T A G T C A G T C A C T G A G T C A G T C A G T C | 1e-24 | -56.633160 | 32.58% | 19.87% | motif file (matrix) |
| 3 | 0.633 | C G T A A C T G A C T G C T A G A C T G G T A C A C T G A C T G A C T G A C G T A C G T A G T C | 1e-6 | -15.713902 | 0.50% | 0.02% | motif file (matrix) |
